# Identification and Analysis of Genes Involved in Double Fertilization in Rice

**DOI:** 10.3390/ijms222312850

**Published:** 2021-11-27

**Authors:** Li You, Li Yu, Ronghong Liang, Ruhao Sun, Fan Hu, Xiaoyun Lu, Jie Zhao

**Affiliations:** State Key Laboratory of Hybrid Rice, College of Life Sciences, Wuhan University, Wuhan 430072, China; lilyou@whu.edu.cn (L.Y.); 2012102040065@whu.edu.cn (L.Y.); liangrh@whu.edu.cn (R.L.); sunruhao@whu.edu.cn (R.S.); FanyHu@whu.edu.cn (F.H.); luxiaoyun@whu.edu.cn (X.L.)

**Keywords:** transcriptome, double fertilization, pollen tube guidance, rice

## Abstract

Double fertilization is a key determinant of grain yield, and the failure of fertilization during hybridization is one important reason for reproductive isolation. Therefore, fertilization has a very important role in the production of high-yield and well-quality hybrid of rice. Here, we used RNA sequencing technology to study the change of the transcriptome during double fertilization with the help of the mutant *fertilization barrier* (*feb*) that failed to finish fertilization process and led to seed abortion. The results showed that 1669 genes were related to the guided growth of pollen tubes, 332 genes were involved in the recognition and fusion of the male–female gametes, and 430 genes were associated with zygote formation and early free endosperm nuclear division. Among them, the genes related to carbohydrate metabolism; signal transduction pathways were enriched in the guided growth of pollen tubes, the genes involved in the photosynthesis; fatty acid synthesis pathways were activated by the recognition and fusion of the male–female gametes; and the cell cycle-related genes might play an essential role in zygote formation and early endosperm nuclear division. Furthermore, among the 1669 pollen tube-related genes, it was found that 7 *arabinogalactan proteins* (*AGPs*), 1 *cysteine-rich peptide* (*CRP*), and 15 *receptor-like kinases* (*RLKs*) were specifically expressed in anther, while 2 *AGPs*, 7 *CRPs*, and 5 *RLKs* in pistil, showing obvious unequal distribution which implied they might play different roles in anther and pistil during fertilization. These studies laid a solid foundation for revealing double fertilization mechanism of rice and for the follow-up investigation.

## 1. Introduction

In angiosperms, female gamete is embraced in the multicellular ovule, while the male gametes produce in another tissue-anther. To enable sexual reproduction, the nonmotile sperm cells are delivered to the female gametophyte (embryo sac) by the elongated pollen tube. This is a long and complex process, and is precisely regulated by a range of genes. The pollen tubes germinate at the surface of stigmatic papilla cells, penetrate the stigma cells, extend polarly in the transmitting tract of style, enter one of synergid cells, and rupture and release the two male gametes into embryo sac. Then, one sperm cell fuses with an egg cell to form a fertilized egg cell (zygote), while the other fuses with a central cell with two polar nuclei to form the primary endosperm nucleus, which is called double fertilization in angiosperms [1]. The former develops as an embryo, while the latter forms endosperm, which is the main part of seed in cereal. Therefore, the success of the process plays a crucial role for high grain yield.

During the process, many glycoproteins participate in it and are indispensable for the guided growth of pollen tubes and the double fertilization. It has been found that the extracellular matrix of transmitting tract in higher plant contains a large number of arabinogalactan proteins (AGPs), which are crucial for the growth of pollen tubes in the tissue of plant style passage [2]. In tobacco (*Nicotiana*), a S-RNase, with the help of AGP 120K (120 KDa glycoprotein), is involved in the recognition of pollens and the self-incompatibility [3,4]. The other researchers found that the stylar AGPs, transmitting-tract-specific (TTS) protein, and pistil extensin-like protein III (PELP III), bind S-RNase in vitro and are also known to interact with pollen or pollen tube [3,5]. It was further confirmed that TTS protein can be deglycosylated in pollen tubes, and the degree of glycosylation in style shows a gradient distribution [6]. In *Arabidopsis*, AGP6 and AGP11 are specifically expressed in pollen and pollen tube. The inhibition of their expression leads to pollen abortion, and the abnormal germination and growth of pollen tubes [7,8]. In *Torenia fournieri*, the ovular methyl-glucuronosyl arabinogalactan induces competency of the pollen tubes to respond to the ovular attractant LURE peptides, indicating that it participates in the ovular guidance of the pollen tubes [9]. The other reports show that AGPs can affect the cytosolic oscillatory calcium ion (Ca^2+^) waves by binding and releasing Ca^2+^ at different pH, typically involving exocytosis of AGPs and recycled Ca^2+^, which may induce the responses of different signal pathways to affect the interaction of the pollen tube and the pistil [10,11]. The other genes related to the Ca^2+^ signal pathway also affect the germination and growth of pollen tubes, such as the pollen-specific gene *OsMLO12* (*POWDERY MILDEW RESISTANCE LOCUS O 12*) and the pistil-preferential gene *OsCNGC13* (*Cyclic nucleotide-gated channel 13*) [12,13].

More evidence shows that the communication between pollen tube and pistil is mainly through the receptor-ligand system. When the pollen falls on the stigma, the pollen ligand SP11/SCR (*S*-locus protein 11/*S*-locus cysteine-rich protein) binds to the stigma receptor SRK (*S*-locus receptor kinase) and activates the downstream molecules MLPK (*M*-locus protein kinase) and E3 ligase ARC1 (arm repeat-containing protein 1) to recognize the compatible pollen [14,15,16,17]. As the pollen tube germinates and grows, the pollen tube/pollen-specific ligands AtRALF4/19 (rapid alkalinization factor 4/19), whose functions depend on AtLRXs (leucine-rich repeat extensins), and interact with the BUPS1/2-ANX1/2-LLG2/3 (Buddha’s paper seal 1/2-ANXUR1/2-LORELEI-like-GPI-anchored protein 2/3) receptor complex to maintain the integrity and the guided growth of the pollen tube [18,19,20]. When the pollen tube reaches around the micropyle, many peptides from the female gametophyte attract it, and the knock-out of these peptides leads to the failure of the guided growth and the entry of pollen tube into embryo sac, such as LUREs, ZmEA1 (*Zea mays* Egg apparatus 1), and AtXIUQIU1-4 [21,22,23,24,25]. These peptides are almost specifically expressed in pistil or just in the female gametophyte, and interact with the receptors in pollen tube to activate the downstream signal pathways, for example, AtLURE1 interacts with AtPRK6 (pollen-specific receptor-like kinase 6), AtMDIS1 (male discoverer 1), and AtMIK1/2 (MDIS1-interacting receptor-like kinase 1/2) [22,26]. The pollen tube penetrates the micropylar nucellus region of ovule and enters one synergid cell, and the female gametophyte-specific cysteine-rich peptides (CRPs), such as ZmES4 (embryo sac 4) and AtRALF34, function as signals to induce the burst of pollen tube by interacting with receptors at the pollen tube surface [18,19,27]. ZmES4 interacts with KZM1 (K^+^ channel *Zea mays* 1) to affect the K^+^ influx of pollen tube, while AtRALF34 interacts with the receptor complex BUPS1/2-ANX1/2-LLG2/3 to induce pollen tube rupture [18,19,27]. The synergid cell-specific receptors AtFER (FERONIA) and LRE (LORELEI) accept the signals from the pollen tube and inhibit its growth in the synergid cell [28,29]. With the help of the signals, the sperm cells are released in the synergid cell and transferred into the egg cell and central cell for double fertilization. The egg cell-secreted peptides AtEC1s (egg cell 1) activate the sperm cell and change the distribution of its transmembrane protein AtGCS1/HAP2 (generative cell specific 1/Hapless 2) to promote the fusion of male and female gametes [30,31]. Therefore, with the help of these proteins, especially the receptors and the CRPs, the two sperm cells are delivered into the embryo sac and fuse with the female gametes to finish the double fertilization successfully.

Agriculturally, successful fertilization is the key determinant of grain yield, and the failure of fertilization in hybridization of different rice varieties is one important reason for reproductive isolation. Due to the existence of reproductive isolation between rice subspecies, the hybrid varieties are mainly intra-subspecific hybrids whose heterosis is limited, greatly hindering the cultivation of rice inter-subspecific hybrids with high yield and good quality [32]. Thus, the studies on the molecular mechanism underlying rice fertilization are significant for the utilization of heterosis and the breeding of new rice varieties (hybrids). However, few studies are conducted in the associated filed and just some rice genes are found to participate in it, such as *OsPTB1* (*pollen tube blocked 1*) and *OsCNGC13* [13,33].

In this study, we performed RNA-seq analysis using Nipponbare (Nip) and a mutant named *fertilization barrier* (*feb*) that failed to complete the double fertilization and led to severe seed abortion. We identified 1669 genes that were related to the growth and guidance of pollen tubes, 332 genes that were involved in the recognition and fusion of the male–female gametes, and 430 genes that were associated with zygote formation and early free endosperm nuclear division. We further analyzed their expression patterns and some gene families that might function in the double fertilization. This might shed light on the molecular mechanism of rice double fertilization.

## 2. Results

### 2.1. A Rice Mutant with Fertilization Barrier

Double fertilization is a remarkable feature of green flowering plants, and also a sign that plants enter the sporophyte generation from the gametophyte generation. During the process of the double fertilization (from the germination and elongation of the pollen tube to the recognition and fusion of the male–female gametes), there are precise regulation mechanisms in each step.

**Figure 1:**
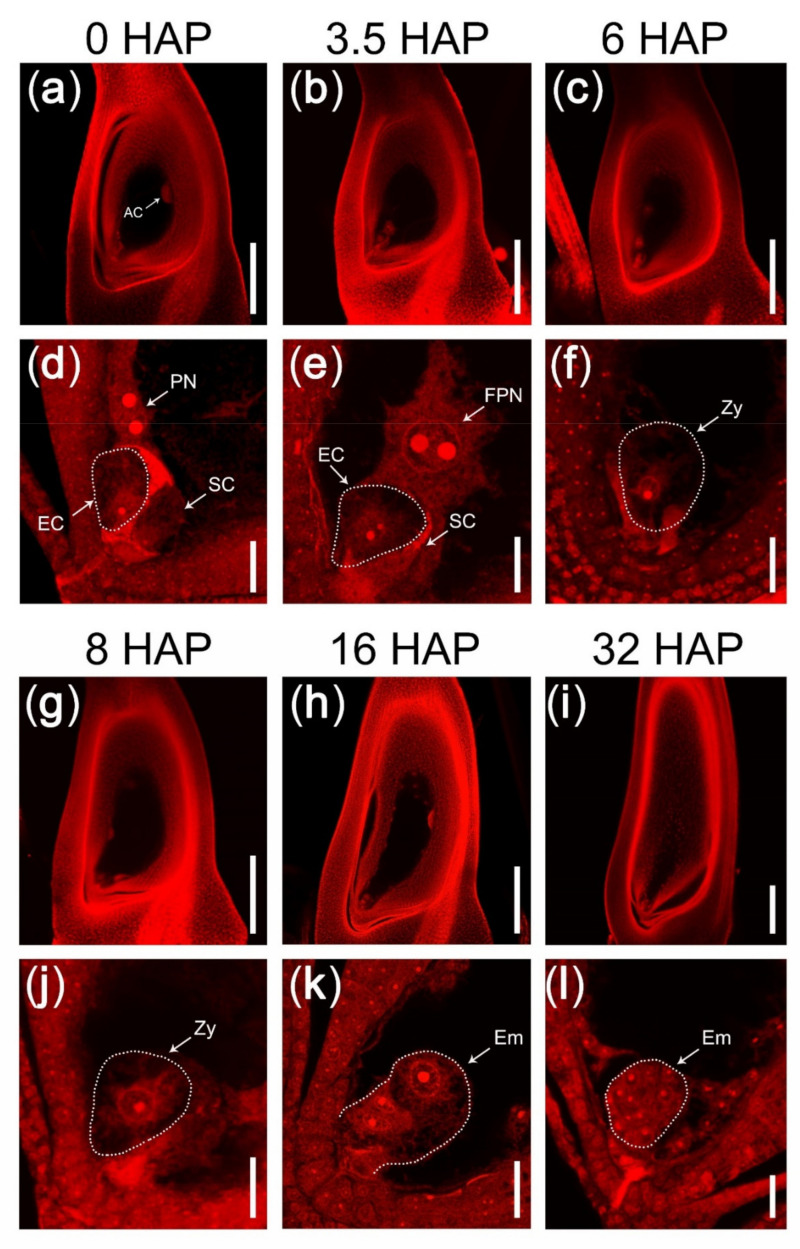
Double fertilization process in rice. (**a**,**d**) A mature embryo sac containing one egg cell (EC), two synergid cells (SC), one central cell with two polar nuclei (PN), and a group of antipodal cells (AC). (**b**,**e**) An embryo sac at 3.5 HAP. The two polar nuclei were fusing and two nucleoli were unfused. (**c**,**f**) A zygote (fertilized egg cell) at 6 HAP. (**g**,**j**) Double fertilization have completed at 8 HAP. (**h**,**k**) The zygote was activated and began to divide at 16 HAP. (**i**,**l**) An early globular embryo with over ten cells at 32 HAP. (**a**–**c**,**g**–**i**) CLSM photos of ovaries stained with eosin B at 10× lens magnification. (**d**–**f**,**j**–**l**) The micropylar part of embryo sacs in (**a**–**c**,**g**–**i**) at 63× lens magnification. The dotted lines indicated the egg cell, the zygote, or embryo. AC, antipodal cell; PN, polar nuclei; FPN, fertilizing polar nuclei; EC, egg cell; SC, synergid cell; Zy, zygote; Em, embryo. Scale bars = 200 μm in (**a**–**c**,**g**–**i**); 20 μm in (**d**–**f**,**j**–**l**).

To study the fertilization mechanisms, we knocked out some pistil-specific genes in Nip and screened out the rice mutants with seed abortion. Luckily, we obtained a mutant named *feb* with severe seed abortion. To observe the causes of abortion, we performed eosin B staining and clear ovary observation on wild-type (WT) and the mutant *feb* using confocal laser scanning microscopy (CLSM). In WT, the mature embryo sac contained two synergid cells and one pear-shaped egg cell at the micropylar end, one large central cell with two apparent polar nuclei in the middle, and a group of antipodal cells at the chalazal end (Figure 1a,d). About one hour after pollination (HAP), several pollen tubes grew towards embryo sac and reached near the micropylar end [33], and only one of them succeeded to enter embryo sac and released two sperm cells. At 3.5 HAP, a sperm cell fused with two polar nuclei to form the primary endosperm nucleus (Figure 1b,e). The primary endosperm nucleus immediately started to divide and evenly attached to the embryo sac wall. At 3.5–6 HAP, the other sperm cell entered the egg cell and fused with it to form the zygote (fertilized egg cell) (Figure 1b,c,e,f). At 8 HAP, four to eight free endosperm nuclei adhered to the embryo sac wall, while the zygote matured (Figure 1g,j). At 16 HAP, the zygote was activated and underwent mitosis to form a 2- to 4-cell pro-embryo (Figure 1h,k). At 32 HAP, the early globular embryo formed, and a large number of free endosperm nuclei were present in embryo sac (Figure 1i,l).

**Figure 2:**
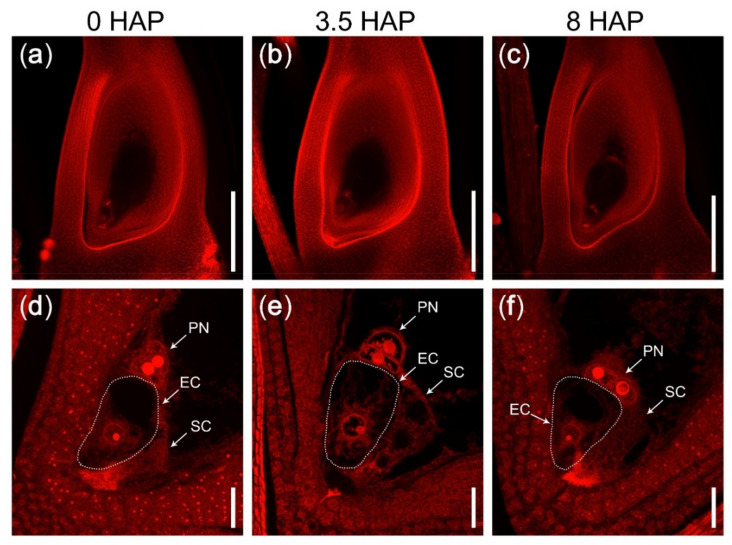
The mutant *feb* failed to complete double fertilization in rice. (**a**,**d**) The mature embryo sac with egg cell and polar nuclei developed normally in the mutant *feb*. (**b**,**c**,**e**,**f**) The mutant embryo sac remained unfertilized at 3.5 HAP (**b**,**e**) and at 8 HAP (**c**,**f**). (**a**–**c**) CLSM photos of ovaries stained with eosin B at 10× lens magnification. (**d**–**f**) The micropylar part of embryo sacs in (**a**–**c**) at 63× lens magnification. The dotted lines indicated the egg cell. EC, egg cell; PN, polar nuclei; SC, synergid cell. Scale bars = 200 μm in (**a**–**c**); 20 μm in (**d**–**f**).

In the mutant *feb*, the plants grew normally and had no obvious difference compared with WT plants during vegetative development. With the help of CLSM, we found that the mature embryo sac of the mutant *feb* also developed normally (Figure 2a,d). However, at 3.5 HAP and 8 HAP, the central cell and the egg cell were unable to fuse with the male gametes to complete the double fertilization, and the polar nuclei always existed as the form of two unfused nuclei (Figure 2b,c,e,f). At 16 HAP and thereafter, most of the embryo sacs remained unfertilized, while a few were fertilized, eventually leading to severe seed abortion.

### 2.2. Strategy to Identify Genes Related to Rice Double Fertilization Process

**Figure 3:**
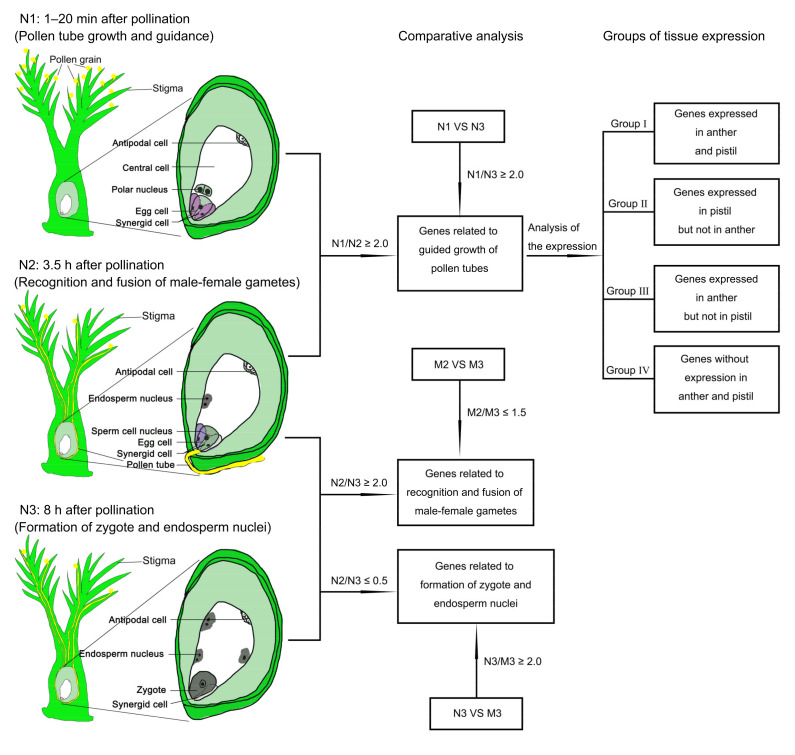
Strategy to identify genes related to rice pollen tube guidance and double fertilization. N1, WT pistil at 1–20 min after pollination; N2, WT pistil at 3.5 HAP; N3, WT pistil at 8 HAP; M1, the mutant pistil at 1–20 min after pollination; M2, the mutant pistil at 3.5 HAP; M3, the mutant pistil at 8 HAP.

In order to identify the changes of gene expression involved in different key biological events during double fertilization, we performed RNA sequencing (RNA-seq) using the pistils of rice wild-type (Nip) and the mutant *feb*. Based on the double fertilization process described above, we collected pistils at three stages, WT (Nip) and mutant pistils at 1–20 min after pollination as samples N1 and M1 (the pollen tubes were germinating or growing), WT and mutant pistils at 3.5 HAP as samples N2 (the fusion of male and female gametes was going on) and M2 (unfertilized), and WT and mutant pistils at 8 HAP as samples N3 (the double fertilization had been finished) and M3 (unfertilized), respectively. Each sample had three biological replicates to ensure the reliability of the RNA-seq results. Then, we conducted comparative analysis of the gene expression profiles of WT and mutant pistils at three stages to identify the changes of gene expression responding to different biological processes. The pollen tube-related genes (PTRGs) were highly expressed in N1 while lowly expressed in N2 and N3, and N1-preferential genes were identified, which were significantly downregulated in both N2 and N3 (N1/N2 ≥ 2, N1/N3 ≥ 2, and *p*-adjust < 0.05), and they might be involved in the growth and guidance of pollen tubes. Since the N1 sample contained pistil and pollen grains adhering to stigmas, we further divided these genes into four groups (Group I–IV) according to the expressions in anther and pistil, including the genes expressed in both pistil and anther (Group I), the genes expressed in pistil but not in anther (Group II), the genes expressed in anther but not in pistil (Group III), and the genes without expression in pistil and anther (Group IV), which might be induced by the germination and/or growth of pollen tubes. Since the mutant *feb* failed to complete the fusion of the male–female gametes, we obtained the genes associated with the recognition and fusion of the male–female gametes through identifying the genes that were significantly downregulated in N3 compared with N2 (N2/N3 ≥ 2 and *p*-adjust < 0.05), but not downregulated or slightly downregulated in M3 compared with M2 (M2/M3 ≤ 1.5). Finally, we obtained the genes related to the zygote formation and the endosperm nuclear division through identifying the genes that were significantly upregulated in N3 compared with N2 (N2/N3 ≤ 0.5 and *p*-adjust < 0.05), and significantly downregulated in M3 compared with N3 (N3/M3 ≥ 2 and *p*-adjust < 0.05) (Figure 3).

### 2.3. Global Analysis of the RNA-seq Data

Three independent cDNA libraries with 150 bp fragments were constructed for each sample, and more than 50 million clean reads were obtained for each library. We downloaded rice genome data and gene models from the RGAP (Rice Genome Annotation Project) website (http://rice.uga.edu/, accessed on 23 November 2021) and assembled the clean reads on them. The results showed that over 97% of the clean reads for each sample were mapped to the reference genome, and 92.50% to 94.52% of them were uniquely mapped (Table S1).

To normalize gene expression level, the FPKM (fragments per kilobase of transcript per million mapped reads) value of each gene was calculated, and the genes with FPKM values lower than 0.5 were considered to be unexpressed genes. Furthermore, to ensure the sequencing data were saturated, we performed sequencing saturation analysis on all the samples. The results showed that the genes with FPKM > 0.3 were nearly saturated when the resampling rates were more than 95% (Figure S1), indicating the high quality of the overall saturation and the reliability of the sequencing data, especially for the expressed genes (FPKM ≥ 0.5). Meanwhile, it was found that more than 22,000 expressed genes were included in each sample, and shared similar expression distributions (Figure S2a). The FPKM values of most genes ranged from 1 to 100, and the value of the most enriched genes was around 10, indicating the overall similarity of transcriptome among different samples (Figure S2a). Moreover, the hierarchical cluster analysis and the principal component analysis (PCA) were performed to assess the overall relation of the transcriptome from different samples, showing that three biological replicates at the same developmental stage were highly correlated and indicating the repeatability of three biological replicates was reliable (Figure S2b). Interestingly, PCA analysis showed that the locations of both N2 and M3 as well as both N1 and M1 were close (Figure S2c), implying that the samples M1 and M3 were similar to N1 and N2, respectively. The similarity of M1 and N1 indicated that the gene mutation in *feb* did not affect the development of the pistil or the anther. The similarity of M3 and N2 showed that the process of fertilization in *feb* at 8 HAP was at the stage of the recognition and fusion of the male–female gametes like N2, hinting that the recognition and fusion of the male–female gametes in the mutant *feb* was stagnated.

### 2.4. Identification of Pollen Tube-Related Genes

**Figure 4:**
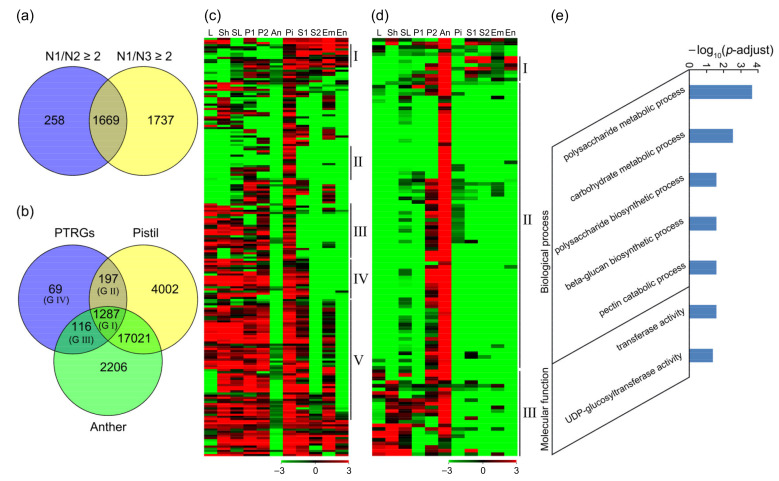
Identification and analysis of the pollen tube-related genes in rice. (**a**) Venn analysis of the 1927 (258 + 1669) genes downregulated in N2 and the 3406 (1669 + 1737) genes downregulated in N3, both compared with N1. (**b**) Venn analysis of the 1669 pollen tube-related genes. Among the 1669 N1-preferential genes, there were 1287 genes (Group I, G I) expressed in both pistil and anther, 197 genes (Group II, G II) expressed in pistil but not in anther, 116 genes (Group III, G III) expressed in anther but not in pistil, and 69 genes (Group IV, G IV) not expressed in pistil and anther. (**c**) Heat map of the 197 pistil-specific genes. (**d**) Heat map of the 116 anther-specific genes. (**e**) GO enrichment analysis of the 116 anther-specific genes. N1, WT pistil at 1–20 min after pollination; N2, WT pistil at 3.5 HAP; N3, WT pistil at 8 HAP; PTRGs, pollen tube-related genes; L, 20-day-old leaf; Sh, 14-day-old seedling on the ground (shoot); SL, seedling at four-leaf stage; P1, panicle before emerging from the sheath of the flag leaf; P2, panicle after emerging from the sheath of the flag leaf; An, mature anther; Pi, mature pistil before pollination; S1, seed at 5 days after pollination (DAP); S2, seed at 10 DAP; Em, embryo at 25 DAP; En, endosperm at 25 DAP.

In flowering plants, the immobile sperm cells are delivered to the female gametophyte to finish double fertilization through transmission of pollen tubes. The guided growth of pollen tubes requires extensive communications with the pistil, and their underlying signaling mechanisms are very complicated, which are rarely dissected in rice. As described above, rice pollen tubes germinate after landing on the stigma and invade the transmitting tissue of style during 1–20 min after pollination [33]. Here, we found that the pollen tubes had reached the embryo sac at 3.5 HAP and the double fertilization had been finished at 6 HAP. In order to obtain pollen tube-related genes, we performed the comparative analysis of samples N1 vs. N2 and N1 vs. N3, and identified 1927 and 3406 N1-preferential genes, respectively, with fold change ≥ 2 and *p*-adjust < 0.05. Among them, 1669 genes were downregulated in both N2 and N3 compared with N1 (Figure 4a). GO (gene ontology) enrichment analysis showed that a total of 79 terms (gene categories) were significantly enriched (*p*-adjust < 0.05) (Table S2). For biological process, three cell wall-related terms, two pectin-related terms, and 12 carbohydrate-related terms were enriched, indicating that the growth of pollen tubes might destroy the cell wall, whose degradation was required to allow the pollen tubes to pass through layers of cells. On the other hand, the pollen tubes might communicate with the pistil through cell walls and their extracellular matrices to maintain their normal growth rate and direction. In addition, pectin and various carbohydrates could provide energy and substances for the growth of pollen tubes. What’s interesting was that hormones were also involved in the pollen tube growth. There were six enriched terms, especially three auxin-related terms, but their function needed further studies. Moreover, six kinds of genes related to response to external signals and six kinds of genes related to substance transport were also enriched, indicating that they were necessary for pollen tube to conduct signal and substance communications with pistil and respond to them all the time, and adjusting the growth direction and state of pollen tube to ensure its arrival at embryo sac (Table S2). For cell component, these genes were almost only enriched in cell wall and extracellular region, where the interactions of pollen tube with pistil took place (Table S2). For molecular function, many enzyme-related genes were enriched, which might be because the growth of pollen tubes required the degradation of cell wall, as well as a large amount of substance and energy. Interestingly, Ca^2+^-related genes were also abundant, implying that the concentration and gradient distribution of Ca^2+^ were necessary for the germination and guided growth of pollen tubes. The downstream signal components of Ca^2+^, such as calmodulin, were also enriched. Besides, there were some transcription factors with transcription repression activity (Table S2). Furthermore, KEGG (Kyoto Encyclopedia of Genes and Genomes) enrichment analysis revealed that three carbohydrate metabolism pathways, one lipid metabolism pathway, and one hormone signal transduction pathway were significantly enriched (Table S3), which confirmed the results of GO enrichment analysis.

In order to explore the different roles that the pistil-expressed genes and the stamen-expressed genes played in the growth of pollen tubes, we downloaded the gene expression data in various rice tissues from RGAP, analyzed the expression patterns of the PTRGs, and used FPKM ≥ 0.5 as the standard for the expressed genes. Thus, 1669 PTRGs were further divided into four groups: 1287 genes expressed in both pistil and anther (Group I), 197 genes expressed in pistil but not in anther (Group II), 116 genes expressed in anther but not in pistil (Group III), and 69 genes not expressed in both pistil and anther (Group IV) (Figure 4b).

In Group I with 1287 genes presented in both pistil and anther (Figure S3, Table S4), 100 genes were predominantly expressed in the panicle after emerging from the sheath of the flag leaf (P2), the anther, and the pistil, which might be closely related to the maturation of pistil and anther (Figure S3a); 81 genes were not expressed in seed at 10 days after pollination (DAP), embryo, and endosperm at 25 DAP, implying that these genes were not involved in the late development of seeds (Figure S3b); 149 genes were not expressed in seed at 10 DAP and endosperm at 25 DAP (Figure S3c), and 41 genes were not expressed in embryo at 25 DAP (Figure S3d), indicating they might function in embryo or endosperm, respectively. However, most of the rest genes were widely expressed in vegetative and reproductive tissues, which were presumably involved in cell basic metabolism (Figure S3e). GO enrichment analysis was similar to that of 1669 PTRGs. The differences were that six signal transduction-related terms were significantly enriched, but there were no auxin-related terms in only two hormone-related terms (Table S5). KEGG enrichment analysis showed that these genes were enriched in one signal transduction pathway, three carbohydrate metabolism pathways, one carotenoid biosynthesis pathway, one lipid metabolism pathway, and one amino acid metabolism pathway (Table S6).

In Group II, 197 genes were expressed in pistil but not in anther (pistil-specific genes) (Table S7). Analysis of the expression patterns revealed that 11 genes were predominantly expressed in pistil and seed, suggesting that these genes might be involved in double fertilization and seed development, respectively (Figure 4c(I)); 16 genes were preferentially expressed in pistil (Figure 4c(II)); 25 genes were highly expressed in vegetative tissues, panicles and pistil, but not in seeds (Figure 4c(III)), implying these genes might participate in the development of the pistil; 19 genes were predominantly expressed in vegetative tissues, panicles, pistil, and seed at 5 DAP, but not in late developed seeds (Figure 4c(IV)); 57 genes were highly expressed in other tissues except anther, seed at 10 DAP, and endosperm at 25 DAP, implying that these genes might participate in double fertilization and embryo development (Figure 4c(V)). GO enrichment analysis showed that five hormone-related terms, nine peptidase-related terms, and two cell wall-related terms were enriched (Table S8), implying that the hormones in pistil were closely related to the guided growth of pollen tubes, and that many small peptides in pistil might play great roles in signal communication between pollen tube and pistil.

In Group III, 116 genes were expressed in anther but not in pistil (anther-specific genes) (Table S9), and the heat map showed that there were anther-specific expression patterns in most of the genes, which were further divided into three clusters (Figure 4d). In these genes, there were 7 genes expressed in P2, anther, and seed (Figure 4d(I)), which might be involved in seed development; 80 genes only expressed in P2 and anther or specifically expressed in anther (Figure 4d(II)), implying that these genes were likely to have important functions in pollen or pollen tube; 24 genes expressed in vegetative tissues, panicle, and anther, but not in pistil and seed (Figure 4d(III)). GO enrichment analysis revealed that four carbohydrate-related terms and one pectin-related term were significantly enriched for biological process, suggesting that anther-specific genes might participate in carbohydrate and pectin metabolism in pollen tube and provide energy for forming the pollen tube wall. For molecular function, two transferase-related terms were enriched, indicating that the germination and growth of pollen tubes required a large amount of substance synthesis (Figure 4e, Table S10). Furthermore, KEGG enrichment analysis showed that one carbohydrate metabolism pathway and one lipid metabolism pathway were enriched (Table S11).

**Figure 5:**
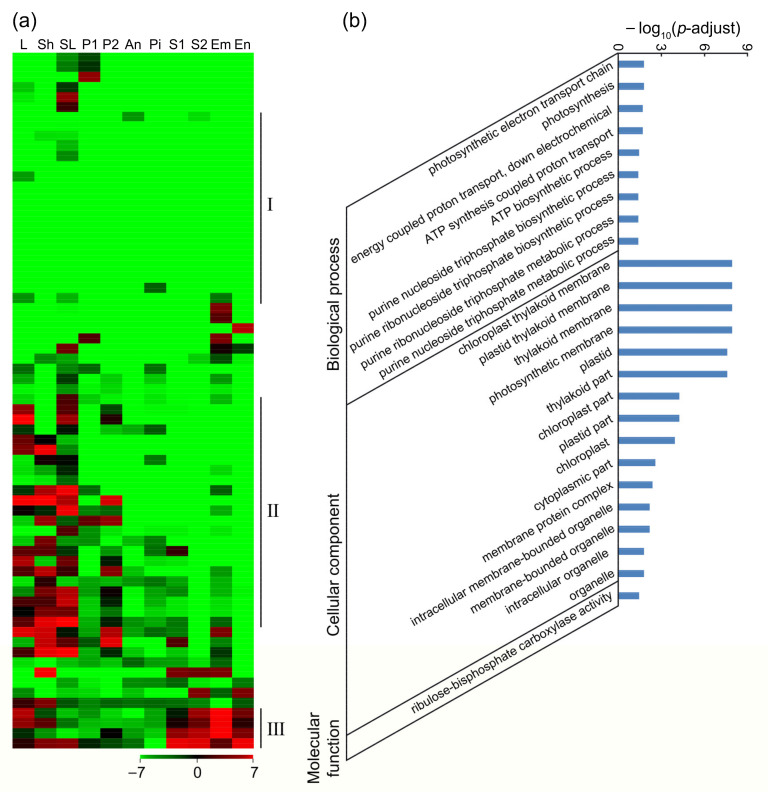
Analysis of the 69 genes (Group IV) induced by the germination or growth of pollen tubes in rice. Heat map (**a**) and GO enrichment analysis (**b**) of the 69 genes induced by the germination or growth of pollen tubes. L, 20-day-old leaf; Sh, 14-day-old seedling on the ground (shoot); SL, seedling at four-leaf stage; P1, panicle before emerging from the sheath of the flag leaf; P2, panicle after emerging from the sheath of the flag leaf; An, mature anther; Pi, mature pistil before pollination; S1, seed at 5 DAP; S2, seed at 10 DAP; Em, embryo at 25 DAP; En, endosperm at 25 DAP.

What is interesting was that in Group IV, 69 genes were not expressed in pistil and anther, but expressed during the growth of pollen tubes (Table S12), suggesting that they might be activated via the guided growth of pollen tubes. In these genes, 19 genes were not expressed in all the queried tissues, suggesting that they might be induced by the growth of pollen tubes (Figure 5a(I)); 23 genes were mainly expressed in the vegetative tissues, and 9 of them were expressed in panicle, but not in other reproductive tissues (Figure 5a(II)); and 4 genes were highly expressed in vegetative tissues and seeds (Figure 5a(III)). GO enrichment analysis showed that a large number of chloroplast-localized photosynthesis-related genes and ATP synthesis-related genes were significantly enriched (Figure 5b, Table S13), implying that photosynthesis process became stronger in pistil after pollination. This might be because the growth of pollen tubes needed to synthesize and consume a lot of ATPs. KEGG enrichment analysis revealed that the genes related to the photosynthesis pathway and the oxidative phosphorylation pathway were significantly enriched (Table S14), verifying that the chloroplast-located genes were highly expressed in the pollinated pistil and the photosynthesis was enhanced after pollination.

### 2.5. Distribution of AGP, CRP, and RLK Genes during the Guided Growth of Pollen Tubes

**Figure 6:**
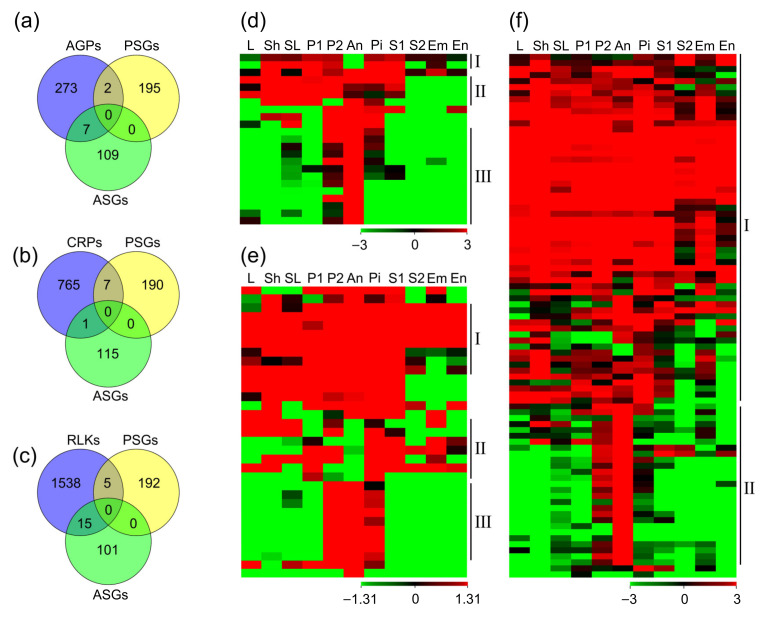
Venn analysis and heat map of *AGP*, *CRP*, and *RLK* genes in rice. (**a**) Among the 282 *AGPs* in rice, two genes were pistil-specific, and seven genes were anther-specific. (**b**) Among the 773 *CRPs* in rice, seven genes were pistil-specific, and one gene was anther-specific. (**c**) Among the 1558 *RLKs*, five genes were pistil-specific, and 15 genes were anther-specific. (**d**) Heat map of the 23 pollen tube-related *AGPs*. (**e**) Heat map of the 33 pollen tube-related *CRPs*. (**f**) Heat map of the 89 pollen tube-related *RLKs*. PSGs, pistil-specific genes that were expressed in pistil but not in anther; ASGs, anther-specific genes that were expressed in anther but not in pistil; L, 20-day-old leaf; Sh, 14-day-old seedling on the ground (shoot); SL, seedling at four-leaf stage; P1, panicle before emerging from the sheath of the flag leaf; P2, panicle after emerging from the sheath of the flag leaf; An, mature anther; Pi, mature pistil before pollination; S1, seed at 5 DAP; S2, seed at 10 DAP; Em, embryo at 25 DAP; En, endosperm at 25 DAP.

As mentioned above, through GO enrichment analysis of the PTRGs, we found that the carbohydrate-related genes preferred to be expressed in anther but not in pistil, while the peptidase-related genes were opposite, implying that carbohydrate factors, such as AGPs and peptides, especially CRPs, might be different in expression patterns and perform different functions in the growth of pollen tubes. Thereupon, we conducted expression analysis of both *AGP* and *CRP* genes. It was reported that there were 282 *AGP* genes in rice [34,35,36], 23 of which belonged to the PTRGs and might take part in the guided growth of pollen tubes (Table S15). Among them, 2 genes were expressed in most of the tissues except anther (Figure 6d(I)); 4 genes were highly expressed in most tissues except seed at 10 DAP, and embryo and endosperm at 25 DAP (Figure 6d(II)); 13 genes were specifically or dominantly expressed in anther, but not or lowly expressed in pistil or other tissues (Figure 6d(III)). Most of the *AGP* genes exhibited higher expression levels in anther than in pistil, and 7 of them were specifically expressed in anther (Figure 6a,d, Table S16), suggesting that the *AGP* genes derived from anther might play more important roles in the formation of pollens and the guided growth of pollen tubes.

CRPs in rice had been predicted [37,38], and there was a total of 773 *CRP* genes that were identified from the RGAP database, 33 of which belonged to the PTRGs (Table S17). Most *CRP* genes were expressed in both pistil and anther, 8 genes were ubiquitously expressed in various tissues (Figure 6e(I)), and 9 genes were only expressed in P2, anther and pistil (Figure 6e(III)). Moreover, we found that 7 genes were not expressed in anther (Figure 6e(II)), while only 1 gene was not expressed in pistil (Figure 6b,e, Table S16), suggesting that CRPs in pistil might be more important during fertilization. As reported before, CRPs generally function as ligands in plants and bind to the corresponding receptors alone or to transmit signals with the assistance of other factors [1]. When the pollen tube grows toward the embryo sac, these pistil-preferential CRPs are likely to act as extracellular signaling molecules to interact with the receptors in pollen tube, and then guide the growth direction of the pollen tube [39]. Therefore, as the key receptors of CRPs, RLKs (receptor-like kinases) should be more abundant in anther and pollen tube. As previously reported, RLKs and their subfamily in rice have been identified [40,41,42,43,44]. In our results, the duplicates or genes not present in RGAP 7.0 vision were removed, and a total of 1558 *RLK* genes were identified, 89 of which belonged to PTRGs (Table S18). Analysis of their expression patterns revealed that most genes were widely expressed in various tissues (Figure 6f(I)), and 27 genes were specifically or predominantly expressed in anther, 15 of which were not expressed in pistil (Figure 6f(II)). In contrast, there were only 5 genes that were not expressed in anther (Figure 6c,f, Table S16). Therefore, we speculated that the RLKs preferred to function in anther during fertilization, and constructed the signal channel between the pollen tube and pistil with the CRPs in pistil.

### 2.6. Identification of Genes Involved in the Recognition and Fusion of the Male–Female Gametes

**Figure 7:**
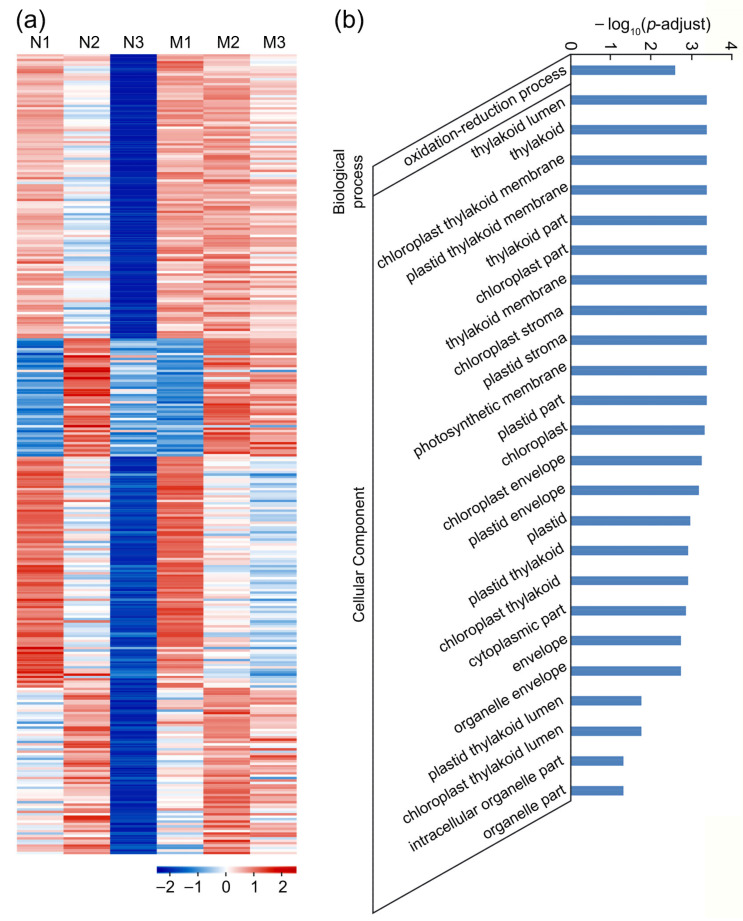
Expression patterns and GO enrichment analysis of the 332 genes involved in the recognition and fusion of the male–female gametes in rice. (**a**) Heat map of the 332 genes, showing significantly downregulated in N3 compared with N2, but not downregulated or slightly downregulated in M3 compared with M2. (**b**) GO enrichment analysis of the 332 genes. N1, WT pistil at 1–20 min after pollination; N2, WT pistil at 3.5 HAP; N3, WT pistil at 8 HAP; M1, the mutant pistil at 1–20 min after pollination; M2, the mutant pistil at 3.5 HAP; M3, the mutant pistil at 8 HAP.

The period in three to four hours after pollination is critical for sperm cells to enter embryo sac and complete double fertilization. We identified 764 genes that were significantly downregulated in N3 compared with N2 (N2/N3 ≥ 2, *p*-adjust < 0.05) in WT and might participate in the fertilization. Among these genes, 332 of them were not downregulated or slightly downregulated in M3 compared with M2 (M2/M3 ≤ 1.5) in *feb* that remained unfertilized at 3.5 and 8 HAP (Figure 7a, Table S19), which might be involved in the recognition and fusion of the male–female gametes. GO enrichment analysis showed that 25 terms were significantly enriched (Figure 7b, Table S20). For biological processes, only genes related to oxidation-reduction process were enriched, implying the recognition and fusion of the male–female gametes needed lots of energy. For the cellular component, most of these genes were located in cell membrane, plastid membrane, and thylakoid membrane, indicating that membrane fusion during double fertilization was mainly determined by membrane-located proteins, which assisted the fusion of the nuclear membranes of the male gamete and the female gamete. Interestingly, many chloroplast-located genes were enriched, which were significantly downregulated in N3 compared with N2, indicating that the photosynthesis in pistil was vital during the fusion and drastically reduced after double fertilization (Figure 7a,b). KEGG enrichment analysis also showed that genes related to photosynthesis and fatty acid biosynthesis were significantly enriched (Table S21), the latter of which were relevant to membrane formation. The expression profiles of these 332 genes in various tissues showed that most genes were widely expressed in vegetative and reproductive tissues, which might participate in basic metabolism such as fatty acid synthesis and photosynthesis (Figure S4a), and that 21 genes were mainly expressed in pistil and seed at 5 DAP, which might be involved in the double fertilization and early seed development (Figure S4b). Moreover, in these 332 genes, there were 12 RLKs and 6 peroxidases (Table S19). The expression analysis showed that 8 RLKs and 4 peroxidases were dominantly expressed in pistil, which was different from the RLKs functioning in the guided growth of pollen tubes. Among them, 6 RLKs and 3 peroxidases were not expressed in anther, hinting that these genes might function in the embryo sac to assist the recognition and fusion of the male–female gametes.

### 2.7. Identification of Genes Involved in Zygote Formation and Endosperm Nuclear Division

**Figure 8:**
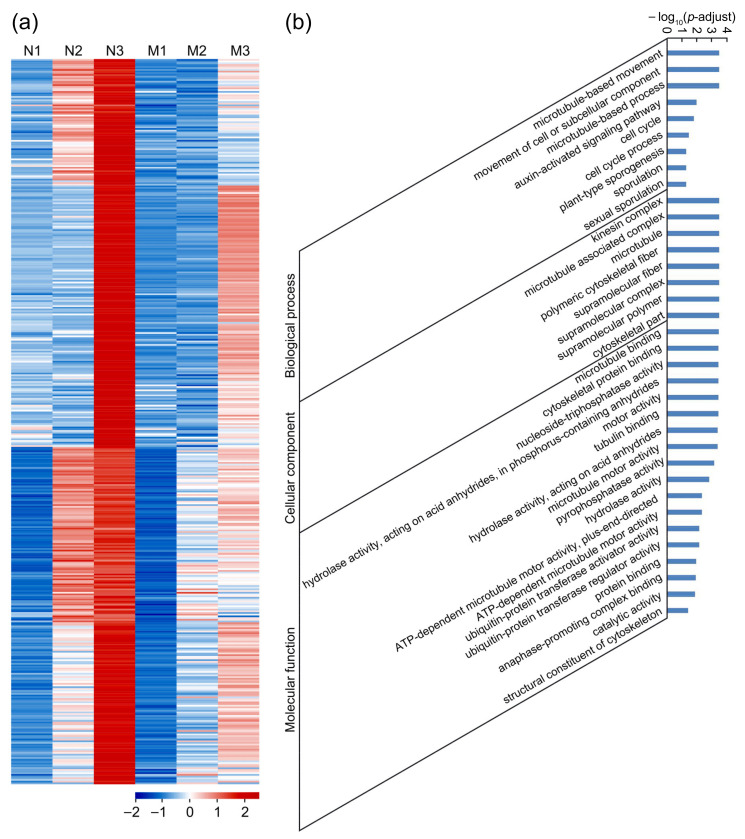
Expression patterns and GO enrichment analysis of the 430 genes involved in zygote formation and free endosperm nuclear division in rice. (**a**) Heat map of the 430 genes, showing significantly upregulated in N3 compared with N2, and significantly downregulated in M3 compared with N3. (**b**) GO enrichment analysis of the 430 genes. N1, WT pistil at 1–20 min after pollination; N2, WT pistil at 3.5 HAP; N3, WT pistil at 8 HAP; M1, the mutant pistil at 1–20 min after pollination; M2, the mutant pistil at 3.5 HAP; M3, the mutant pistil at 8 HAP.

In WT, the zygote had been formed, and the free endosperm nuclei were undergoing vigorous division at 8 HAP, but in the mutant *feb*, the embryo sac was still unfertilized and contained one egg cell and two polar nuclei. The 430 genes we identified were significantly upregulated in N3 compared with N2 (N2/N3 ≤ 0.5, *p*-adjust < 0.05) and had a higher expression level in N3 than in M3 (N3/M3 ≥ 2, *p*-adjust < 0.05) (Table S22), which might be involved in the early development of zygote and the division of endosperm nuclei (Figure 8a). GO enrichment analysis uncovered that a total of 35 terms were significantly enriched (Figure 8b, Table S23). Microtubule, sporulation, and cell cycle-related genes were enriched, implying that the development of zygote and endosperm nuclei required the microtubules. Microtubules functioned in the maturation of zygote before division, and affected the orientation of the cell division plane. Sporulation usually meant unequal material distribution and cell division, hinting that the first round of division of rice zygote was asymmetry. The enrichment of the cell cycle-related genes indicated that the endosperm nuclei were in a rapid division stage, and the cell cycle changed extremely rapidly (Figure 8b). Subsequently, we analyzed the expression data of these genes in various tissues of rice. The results showed that about one fifth of the genes were widely expressed in vegetative and reproductive tissues (Figure S5a); about half of the genes were predominantly expressed in tissues other than seed at 10 DAP and endosperm at 25 DAP (Figure S5b); 25 genes were mainly expressed in P1, pistil and embryo at 25 DAP, indicating that they might be closely related to embryo development.

### 2.8. Validation of Gene Expression Patterns via qRT-PCR

**Figure 9:**
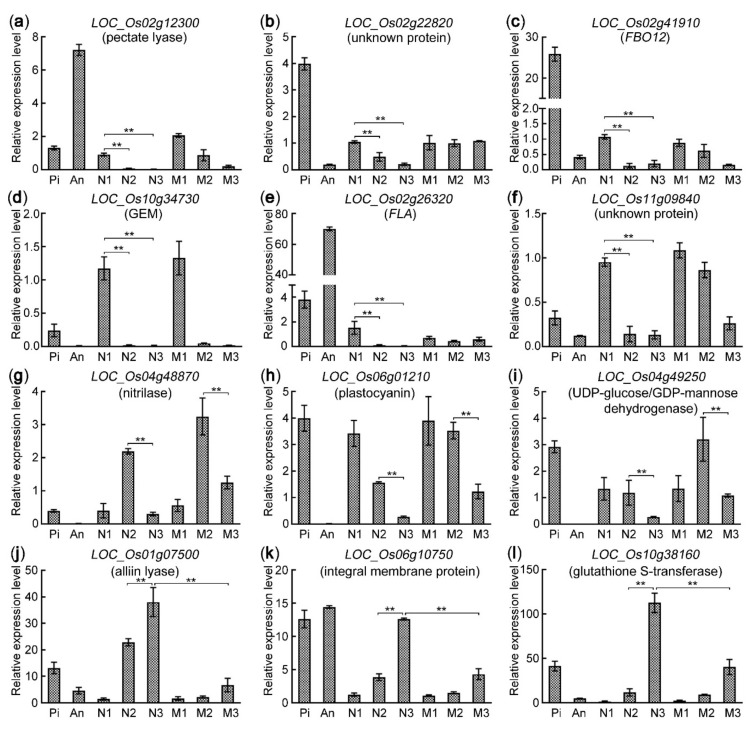
The qRT-PCR validation of gene expression patterns in rice WT and mutant *feb*. (**a**–**f**) The expression patterns of 6 pollen tube-related genes. (**g**–**i**) The expression patterns of 3 genes involved in the recognition and fusion of the male–female gametes. (**j**–**l**) The expression patterns of 3 genes involved in the zygote formation and the free endosperm nuclear division. Three biological replicates and three technical replicates of each sample were performed for qRT-PCR. Two asterisks (**, *p* < 0.01) represent significant differences according to Student’s *t*-test. An, mature anther; Pi, mature pistil before pollination; N1, WT pistil at 1–20 min after pollination; N2, WT pistil at 3.5 HAP; N3, WT pistil at 8 HAP; M1, the mutant pistil at 1–20 min after pollination; M2, the mutant pistil at 3.5 HAP; M3, the mutant pistil at 8 HAP.

In order to validate the reliability of the transcriptome profiles, we used qRT-PCR (quantitative real-time PCR) technique to analyze the expression patterns of 12 genes. The 6 genes of them were preferentially expressed in the sample N1. Among them, *LOC_Os02g12300* (pectate lyase) and *LOC_Os02g22820* (unknown protein) were expressed in both pistil and anther (Figure 9a,b); *LOC_Os02g41910* (FBO12, F-box containing protein) and *LOC_Os10g34730* (GEM, GLABRA 2 expression modulator) were preferentially expressed in pistil (Figure 9c,d); *LOC_Os02g26320* (FLA, Fasciclin-like arabinogalactan protein) was highly expressed in anther but lowly in pistil (Figure 9e); *LOC_Os11g09840* (unknown protein) had lower expression in the mature pistil and the anther, but high in the pollinated pistil (N1) (Figure 9f), hinting that it might be induced by the germination and growth of pollen tubes. *LOC_Os04g48870* (nitrilase), *LOC_Os06g01210* (plastocyanin), and *LOC_Os04g49250* (UDP-glucose/GDP-mannose dehydrogenase) were downregulated in N3 compared with N2 (Figure 9g–i), which might be involved in the recognition and fusion of the male–female gametes. *LOC_Os06g01210* encoded plastocyanin that was located in the chloroplast thylakoid membrane and functioned in the photosynthesis process. *LOC_Os01g07500* (alliin lyase), *LOC_Os06g10750* (integral membrane protein) and *Os10g38160* (glutathione S-transferase) were significantly upregulated in N3 compared with N2 (Figure 9j–l), which might be involved in zygote formation and endosperm nuclear division. All of the qRT-PCR results showed similar gene expression patterns with the transcriptome profiles, confirming the reliability of the RNA-seq data.

## 3. Discussion

### 3.1. The Mutant Feb Could Help to Investigate the Mechanism about the Recognition and Fusion of Male–Female Gametes

In this study, we identified a new mutant *feb* with low seed setting rate and found that there was no abnormality in the vegetative stage. The ovary transparency assay showed that the development of the pistil in *feb* was similar to WT while remained unfertilized at 8 HAP (Figure 1 and Figure 2). Then, the analysis of RNA-seq and the PCA showed that the transcriptome of M1 (1–20 min after pollination in *feb*) was similar to N1 (1–20 min after pollination in WT) while the transcriptome of M3 (8 HAP in *feb*) was similar to N2 (3.5 HAP in WT) (Figure S2c). These results indicated that the development of pistil in *feb* was similar to WT, and there was only one abnormality in *feb* that the recognition and fusion of the male–female gametes was arrested or stagnated at 3.5 and 8 HAP, hinting that *feb* would be a perfect material to investigate the mechanism about the recognition and fusion of the male–female gametes. Using this mutant, we identified 332 genes related to the recognition and fusion of the male–female gametes (Figure S4; Table S19). Unexpectedly, GO and KEGG analysis showed that the genes in photosynthetic pathway and chloroplast were also enriched in these genes (Figure 7b). These might be an interesting point to study the fertilization for no genes about photosynthetic pathway and chloroplast had been confirmed to be related to the recognition and fusion of the male–female gametes. Furthermore, the genes about photosynthetic pathway and chloroplast might provide the energy and regulate the level of reactive oxygen in the embryo sac though no direct evidence has been obtained. When the pollen tube enters the synergid cell, the reactive oxygen promotes the rupture of the pollen tube and inhibits the entry of the other pollen tubes [1]. In *Arabidopsis*, the disruption of mitochondrion ANK6 (Ankyrin repeat protein) or peroxin would affect the recognition and fusion of the male–female gametes [45,46]. In this study, we also found 6 peroxidases functioned in the recognition and fusion of the male–female gametes (Table S19). Besides, 12 RLKs might participate in this process, but only 6 of them were expressed in anther. The 12 genes shared different expression pattern from RLKs that participated in the guided growth of pollen tubes (Figure 6c; Table S19), implying the pistil-specific RLKs might play essential roles in the recognition and fusion of the male–female gametes. In *Arabidopsis*, the FERONIA/FER (a RLK protein), receives the signals from the pollen tube or sperm to promote the fusion of male–female gametes and inhibits the growth of other pollen tubes, and knockout of it will lead to the failure of the male–female gamete fusion and then the entry of multiple pollen tubes to the synergid cell [28]. However, the signal transduction pathway in the recognition and fusion of the male–female gametes was still unclear and needed further research, especially in rice. Therefore, the mutant *feb* would be a very good material to uncover the molecular mechanism.

### 3.2. Photosynthetic Pathway Was Activated in Rice Pistil during the Guided Growth of Pollen Tubes

After the pollen grains fall on the stigma, the pollens germinate and form the tubes, and deliver the male gametes to the embryo sac. During this process, many genes are activated and help to complete fertilization. It is found that these genes are enriched in the metabolism, signal transduction, and cellular transport pathways, and many are located in the cell membrane and the extracellular region which might transduce signals from extracellular environment to the pollen cytoplasm [39,47,48]. Unexpectedly, the transcriptome of rice pistil showed that the genes in photosynthetic pathway and chloroplast were enriched in the 69 pollen tube-activated genes which were not expressed in the mature anther and pistil (Figure 5b). However, no correlation between the pollen tube germination and the photosynthetic pathway was reported. We proposed that there were two possible reasons leading to the result. One was that the growth of pollen tubes needed lots of nutrients and energy [49]. Some of the nutrients could be produced by photosynthesis in pistil, and NADPH-related genes in photosynthesis pathway might affect the concentration of reactive oxygen [50,51]. The other was that, in previous studies, the reactive oxygen was confirmed to function in the germination and growth of pollen tubes in *Arabidopsis* [50,51]. Hence, the reactive oxygen was indispensable for the guided growth of pollen tubes, leading to the activation of genes related to photosynthetic pathway. This inference still needs to be verified by more evidence.

### 3.3. Pistil Communicated with Pollen Tube in Different Species

Rapid and reliable communication between pollen tube and pistil is essential for successful fertilization in angiosperms. The communication between male and female gametophytes is performed in pistil from the adherence of pollen to stigma and to the fertilization in ovule [52]. It is reported that the molecular mechanisms of the development process are mainly regulated through the receptor-ligand system [1]. As the pollen tube germinates and grows, the pollen tube/pollen-specific peptides (ligands) AtRALF4/19 interact with the pollen tube-specific receptor complex AtBUPS1/2-ANX1/2-LLG2/3 to maintain the pollen tube integrity and the guided growth in *Arabidopsis* [18,19]. When the pollen tube enters the micropyle of ovule and penetrates the female gametophyte, the synergid cell-specific peptides AtLUREs interact with AtPRK6, AtMDIS1, and AtMIK1/2 of the pollen tube to attract the guided growth of pollen tube, and the ovule-preferential peptide AtRALF34 competes with AtRALF4/19 for interaction with AtBUPS1 or AtANX1, leading to the burst of the pollen tube and the release of the two sperm cells [18,19,22,26]. In the study of *Papaver rhoeas*, it was found that the polypeptide PrsS (*Papaver rhoeas* stigma *S* determinant) in the stigma cells interacts with receptor PrpS (*Papaver rhoeas* pollen *S*) in the pollen tubes to activate downstream Ca^2+^-dependent signaling pathway, inhibiting the growth of incompatible pollen tubes [53]. These peptides almost are specifically expressed in the female gametophyte and interact with the receptors in the pollen tube to activate the downstream signal pathways. In *Brassicaceae*, there are also some small peptides specifically expressed in pollen/pollen tube. They interact with the pistil-expressed receptors to promote the adhesion and the growth of the pollen tube, like the pollen-expressed peptide SP11/SCR and the pistil-expressed receptor SRK [15]. In our studies, the pistil-specific peptides (ligands) were more than the pollen/pollen tube-specific peptides while the pistil-specific receptors were less. We identified 1669 genes that were related to the growth of pollen tubes and found that 1 *CRP* and 15 *RLKs* were specifically expressed in anther while 7 *CRPs* and 5 *RLKs* in pistil among them (Figure 4a and Figure 6b,c). The distribution of the CRPs and RLKs was similar to the other species, implying that they might have similar mechanisms in rice and need to be further investigated. Hence, we proposed that these CRPs and RLKs might play essential roles during rice fertilization.

### 3.4. AGPs Played Different Roles in Dry and Wet Stigmas

Except the peptides, it is reported that AGPs also play important roles in the adhesion and guided growth of pollen tubes [2]. AGPs are a kind of glycoprotein with high glycosylation and complex structure, and composed of core protein skeleton and huge glycosyl side chain. They widely exist in the tissues and organs of plant kingdom, and can participate in the Ca^2+^ signaling pathway by affecting the cytosolic oscillatory Ca^2+^ waves except that the small AGPs such as the peptides [10,11,54]. The previous studies have shown that AGPs are abundant in the pistils of *Arabidopsis* and *Nicotiana*, and play essential roles during the interaction between the pistil and the pollen/pollen tube [3,4,54]. In the plant with wet stigma (such as tobacco), AGPs exist in the stigma and the transmitting tract tissue to recognize or promote the germination and growth of pollen tubes. In the plant with dry stigma (such as rice and *Arabidopsis*), unlike the wet stigma, no pistil-expressed AGP has been reported in the recognition or promotion of the pollen tube, while AtENODLs (early nodulin-like proteins) are expressed in the micropyle of ovule and function in the reception of the pollen tube [54]. Besides, more AGPs in *Arabidopsis* are expressed in anther and regulate the development of the pollen and the pollen tube, such as AtFLA3 [55] and AtAGP6/11 [56]. In rice, we found that 23 AGPs were involved in the guided growth of pollen tubes. Among them, 7 genes were specifically expressed in anther except 2 genes, implying AGPs might play more important roles in rice anther (Figure 6a). Since AGPs function through their glycosyl side chain, they have high heterogeneity and functional redundancy, which requires the simultaneous knockout of multiple *AGP* genes when studying the detailed functions of multiple *AGP* genes. This might be important for future investigating AGPs roles in rice anther, which would lay the foundation for dissecting the new mechanism of anther development.

### 3.5. This Study Laid Solid Foundation for Investigating Fertilization Mechanism in Rice

In order to dissect the molecular mechanisms of rice fertilization further, we analyzed the transcriptome change in the process of fertilization in Nip and a rice mutant with abnormal fertilization that was named *feb*. The results showed that we identified 1669 genes that were related to the growth of pollen tubes, 332 genes involved in the recognition and fusion of the male–female gametes, and 430 genes involved in zygote formation and early endosperm nuclear division. The GO enrichment analysis showed that many enzyme-related genes, Ca^2+^-related genes, and the genes located in cell wall and extracellular region were enriched in the growth of pollen tubes, and KEGG enrichment analysis indicated that they might participate in the carbohydrate metabolism and hormone signal transduction pathways. Among the genes involved in the recognition and fusion of the male–female gametes, the genes located in the membrane and chloroplast were enriched, suggesting that they might function in photosynthesis and fatty acid synthesis pathways. Microtubule, sporulation, and cell cycle-related genes might play essential roles in the zygote formation and early endosperm nuclear division. Moreover, among the genes related to the growth of pollen tubes, we found that 7 *AGPs*, 1 *CRP*, and 15 *RLKs* were specifically expressed in anther, while 2 *AGPs*, 7 *CRPs*, and 5 *RLKs* in pistil, showing the obvious unequal distribution. These genes might have different functions between pistil and anther, and played essential roles in the double fertilization. In the future, we could knock out these genes to verify our hypothesis and detect the interaction between them and FEB proteins to dissect the molecular mechanism underlying the double fertilization. Thus, this study laid the solid foundation for investigating fertilization mechanism in rice.

## 4. Materials and Methods

### 4.1. Plant Materials and Growth Conditions

Based on the previous research about embryo sac-preferential/specific genes in rice [57], we knocked out *FEB1/2/3* (*Fertilization barrier 1/2/3*, *LOC_Os03g18530*/*LOC_Os11g06730*/*LOC_Os12g06970*) genes using CRISPR/Cas9 technique, which might be predominantly expressed in rice embryo sac and their *Arabidopsis* homologs functioned in the double fertilization. We designed the CRISPR target sites of *FEB1/2/3* on the CRISPRdirect website (http://crispr.dbcls.jp/, accessed on 23 November 2021), and chose the sites closest to ATG to construct CRISPR/Cas9-based multiple genome engineering vector. Then, we transformed the vector into rice Nip (*Oryza sativa* L. ssp. *japonica* cultivar ‘Nipponbare’), and screened out the transgenic plants with the mutations of *FEB1/2/3*, which were named *feb*. The mutant *feb* showed very low seed setting rate, which was caused by the failure of double fertilization.

The rice wild-type variety Nip and the mutant *feb* were used as the test materials, and planted in the green house in Wuhan University at 28 ± 2 °C under long day conditions (14 h light/10 h dark) or grown outdoors under nature conditions. About 80 to 100 pistils were respectively dissected from Nip and the mutant *feb* at 0, 3.5, and 8 HAP for RNA extraction. Pistils from different panicles of the same plant were collected in one microtube as one sample. One pistil per floret and 80–100 florets per plant were collected. And three such microtubes from three different plants with the same stage were used as three biological replicates.

### 4.2. Ovary Clearing and Microscopy Observation

To observe the process of double fertilization in rice ovary, we used a modified WE-CLSM (whole-mount eosin B-staining confocal laser scanning microscopy) protocol based on Zeng’s method [58]. Florets with open glumes were labeled, and collected on ice at different stages. The ovaries were dissected immediately and fixed in FAA for 24 h. Then, the samples were hydrated in 50% ethanol, 30% ethanol, and deionized water for 30 min each step. The ovaries were pretreated in 2% potassium aluminum sulfate for 20 min before staining with 10 mg/L eosin B overnight. Then, the ovaries were treated in 2% potassium aluminum sulfate again for 20 min and rinsed with deionized water three times. The ovaries were dehydrated with a series of ethanol solutions (30%, 50%, 70%, 90%, and 100%), 20 min each step. Finally, they were cleared in ethanol and methyl salicylate (1:1) mixture for 1 h, and then in pure methyl salicylate for 1 h at least. The cleared ovaries were observed under an Olympus FluoView FV1000 confocal laser scanning microscope with a 488-nm excitation laser.

### 4.3. RNA Extraction and Sequencing

Total RNA was isolated and 2 μg of RNA in each sample was treated with DNase I to destroy traces of possible DNA contamination using the TransZol Plant kit (TransGenBiotech, Beijing, China) following the manufacturer’s protocol. Quality of purified RNA was checked by agarose gel electrophoresis and integrity of RNA was checked by RNA 6000 Nano kit (Agilent, Santa Clara, CA, USA). RNA concentration was measured by NanoDrop 2000 spectrophotometer (Thermo Scientific, Waltham, MA, USA). The RNA-seq cDNA libraries were constructed using TruseqTM RNA sample prep kit (Illumina, San Diego, CA, USA). Sequencing was running on an Illumina HiSeq4000 sequencer, and data analyses were performed on Majorbio Cloud platform (Majorbio, Shanghai, China).

### 4.4. Reads Optimization and Transcriptome Assembly

Raw data were saved as fastq format and contained adaptor sequences, uncertain bases, low-quality, and short reads, which should be removed to obtain clean reads for the downstream analyses. Clean reads were mapped to rice reference genome downloaded from RGAP website (http://rice.uga.edu/, accessed on 23 November 2021) using TopHat2 (v2.1.1) with default parameters. Only uniquely mapped reads were spliced together guided by RGAP gene models using Cufflinks (v2.2.1).

### 4.5. Identification of Differentially Expressed Genes and Cluster Analysis

Read counts mapped to the reference genome were calculated by RSEM (v1.3.3). The normalized expression value of each gene was represented by FPKM using Kallisto (v0.46.2). Differentially expressed genes (DEGs) between two samples were identified by DESeq2 (v1.10.1) with fold change ≥ 2 and *p*-adjust < 0.05. *p*-adjust was corrected *p*-value by BH (false discovery rate correction with Benjamini/Hochberg) method. The hierarchical or K-means cluster analyses of expression patterns of DEGs were also performed by DESeq2.

### 4.6. Venn Analysis and Heat Map Construction

Venn analysis of DEGs was performed using VENNY 2.1 (https://bioinfogp.cnb.csic.es/tools/venny/, accessed on 23 November 2021). To draw the heat maps, the data of the second generation transcriptional group were downloaded from the RGAP website (http://rice.uga.edu/expression.shtml, accessed on 23 November 2021), and 11 tissues were used for gene expression analysis, including 20-day-old leaf (L), 14-day-old seedling on the ground (Sh), seedling at four-leaf stage (SL), panicle not emerged from the sheath of the flag leaf (P1), panicle emerged from the sheath of the flag leaf (P2), mature anther (An), mature pistil before pollination (Pi), seed at 5 days after pollination (S1), seed at 10 days after pollination (S2), embryo at 25 days after pollination (Em), and endosperm at 25 days after pollination (En). The expression values of all genes were added with 0.00001, and analyzed by Cluster 3.0 and Treeview (v1.1.6) software, and then the heat map was made [57].

### 4.7. Functional Annotation and Enrichment Analyses

GO analysis of DEGs was performed using Blast2go (v2.5). KEGG analysis of DEGs was performed using KOBAS (v2.1.1). GO terms and KEGG terms with *p*-adjust below 0.05 were considered to be significantly enriched, which were determined by Goatools (v0.6.5) and R script (Majorbio, Shanghai, China), respectively.

### 4.8. Quantitative Real-Time PCR

Before pollination, mature anthers and pistils were collected, respectively. Mature pistils were collected as mentioned above in Section 4.1. Mature anthers were collected from different panicles of the same plant in one microtube as one sample. Anthers from about 50 florets in one plant were used for one sample. Three such microtubes from three different plants with the same stage were used as three biological replicates. Total RNA was isolated and treated with DNase I using the TransZol Plant kit following the manufacturer’s protocol. The reverse transcription was completed with EasyScript One-Step gDNA Removal and cDNA Synthesis SuperMix (TransGenBiotech, Beijing, China). The qRT-PCR was running on the Bio-Rad CFX Connect^TM^ Real-Time System (Bio-Rad, Hercules, CA, USA) in triplicate. PCR amplification was performed in a 15 μL of volume containing 7.5 μL of 2×TransStart Top Green qPCR SuperMix (TransGenBiotech, Beijing, China), 4.5 μL of water, 2 μL of primer (10 μM), and 1 μL of cDNA template. PCR was cycled 40 times as follows: 5 s denaturation at 95 °C, 15 s annealing at 57–60 °C, and 10 s polymerization at 72 °C. Melting curves were checked in the end. Normalized qPCR results were calculated using delta-delta Ct method with the help of the CFX Manager™ (v3.1) software. Error bars were standard deviation (SD) calculated by function STDEV.P in Microsoft Excel. A significance test was performed by Student’s *t*-test in Excel. Two asterisks (**, *p* < 0.01) represent significant differences between the samples. The relative expression value of each gene was calculated as previously reported with *Actin* as an internal control [59]. All primers used in this article were listed in Table S24.

## Data Availability

RNA-seq data of Nip and *feb* pistils have been deposited in NCBI (National Center for Biotechnology Information) under BioProject number PRJNA765935.

## Supplementary Materials

Supplementary Materials can be found at https://www.mdpi.com/article/10.3390/ijms222312850/s1.

## Abbreviations

AGP, arabinogalactan protein; ANX1/2, ANXUR1/2; ARC1, arm repeat-containing protein 1; BUPS1/2, Buddha’s paper seal 1/2; CLSM, confocal laser scanning microscopy; CNGC13, cyclic nucleotide-gated channel 13; CRP, cysteine-rich peptide; DAP, day after pollination; DEG, differentially expressed gene; EC1, egg cell 1; ES4, embryo sac 4; *feb*, *fertilization barrier*; FER, FERONIA; FLA, fasciclin-like arabinogalactan protein; FPKM, fragments per kilobase of transcript per million mapped reads; GCS1/HAP2, generative cell specific 1/Hapless 2; GEM, GLABRA 2 expression modulator; GO, Gene Ontology; HAP, hour after pollination; KEGG, Kyoto Encyclopedia of Genes and Genomes; KZM1, K^+^ channel *Zea mays* 1; LLG2/3, LORELEI-like-GPI-anchored protein 2/3; LRE, LORELEI; LRX, leucine-rich repeat extension; MDIS1, male discoverer 1; MIK1/2, MDIS1-interacting receptor-like kinase 1/2; MLO12, POWDERY MILDEW RESISTANCE LOCUS O 12; MLPK, *M*-locus protein kinase; Nip, Nipponbare; PCA, principal component analysis; PELPIII, pistil extensin-like protein III; PRK6, pollen-specific receptor-like kinase 6; PrpS, *Papaver rhoeas* pollen *S*; PrsS, *Papaver rhoeas* stigma *S* determinant; PTB1, pollen tube blocked 1; PTRG, pollen tube-related gene; RALF4/19, rapid alkalinization factor 4/19; RGAP, Rice Genome Annotation Project; RLK, receptor-like kinase; SP11/SCR, *S*-locus protein 11/*S*-locus cysteine-rich protein; SRK, *S*-locus receptor kinase; TTS, transmitting-tract-specific; WE-CLSM, whole-mount eosin B-staining confocal laser scanning microscopy; ZmEA1, *Zea mays* egg apparatus 1.
