# Supplementary material for: Identification and Analysis of Genes Involved in Double Fertilization in Rice
Source: Int J Mol Sci. 2021 Nov 27;22(23):12850. doi: 10.3390/ijms222312850 (PMC8657449; doi:10.3390/ijms222312850)
Supplement: Supplementary file 1 [file ijms-22-12850-s001.zip › ijms-1479708 sup.pdf]

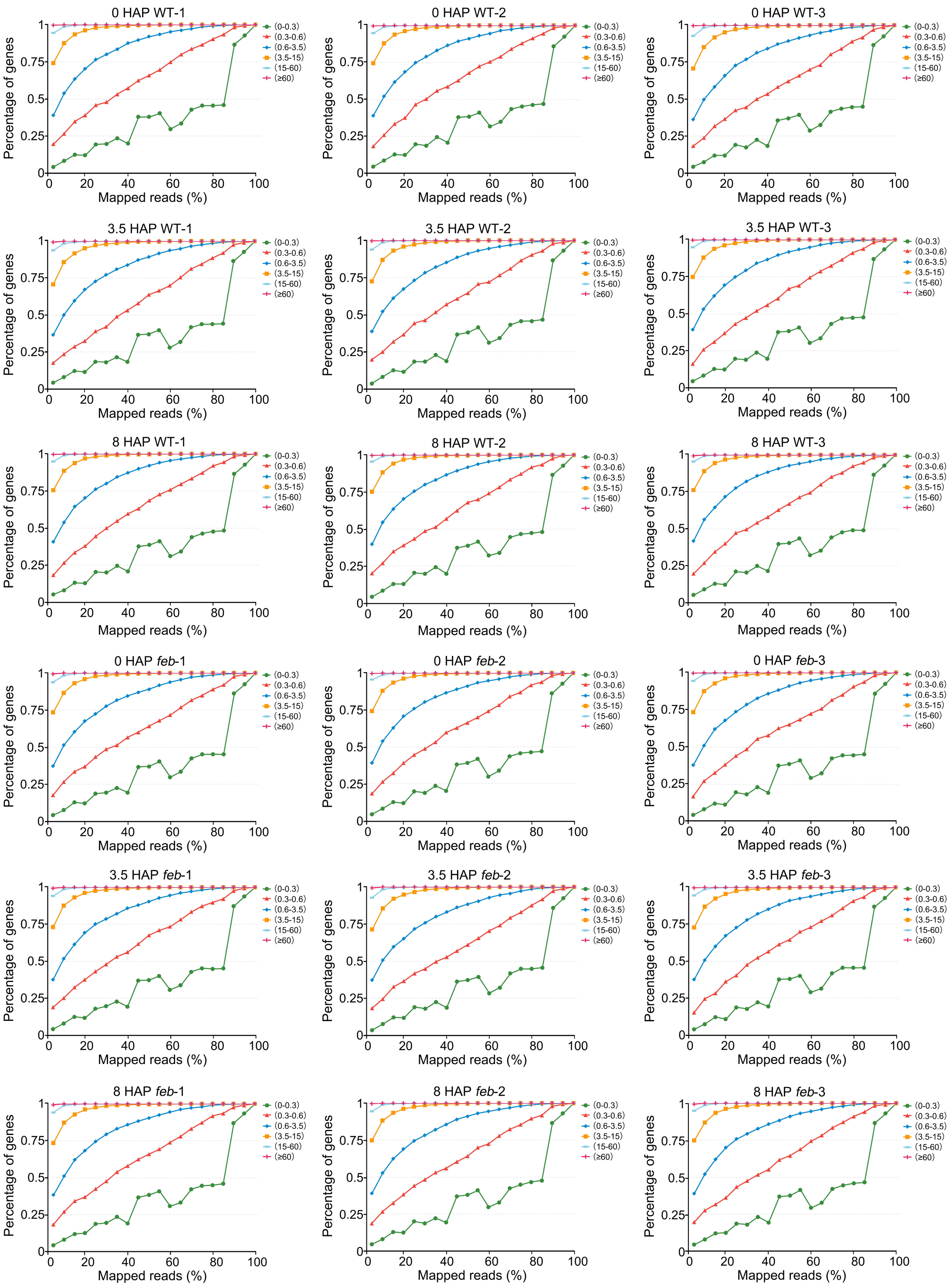

Figure S1. The saturation analysis of all the samples in rice. The abscissa was the percentage of the mapped reads. The ordinate was the percentage of genes with FPKM deviation within 15% of final value. The closer the value was to one, the more saturated the expression level was. Different color lines represented the saturation curves of genes with different expression levels in the samples.

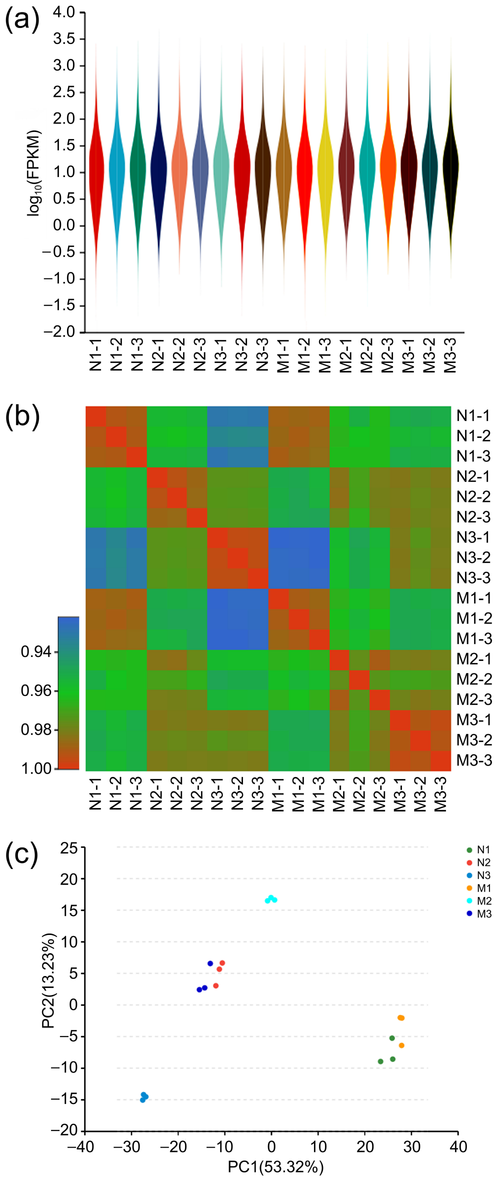

Figure S2. Global analysis of the RNA-seq data. (**a**) Gene expression distribution of each sample. The FPKM value was logarithmically processed by $\log_{10}$. The swollen part represented the region where the gene expression levels of the samples were most concentrated. (**b**) The Pearson correlation coefficients of gene expression between replicates. (**c**) The principal component analysis. The first principal component explained 53.32% of variance, while the second principal component were 13.23% of variance. PC1, the first principal component; PC2, the second principal component; N1, WT pistil at 1–20 min after pollination; N2, WT pistil at 3.5 HAP; N3, WT pistil at 8 HAP; M1, the mutant pistil at 1–20 min after pollination; M2, the mutant pistil at 3.5 HAP; M3, the mutant pistil at 8 HAP.

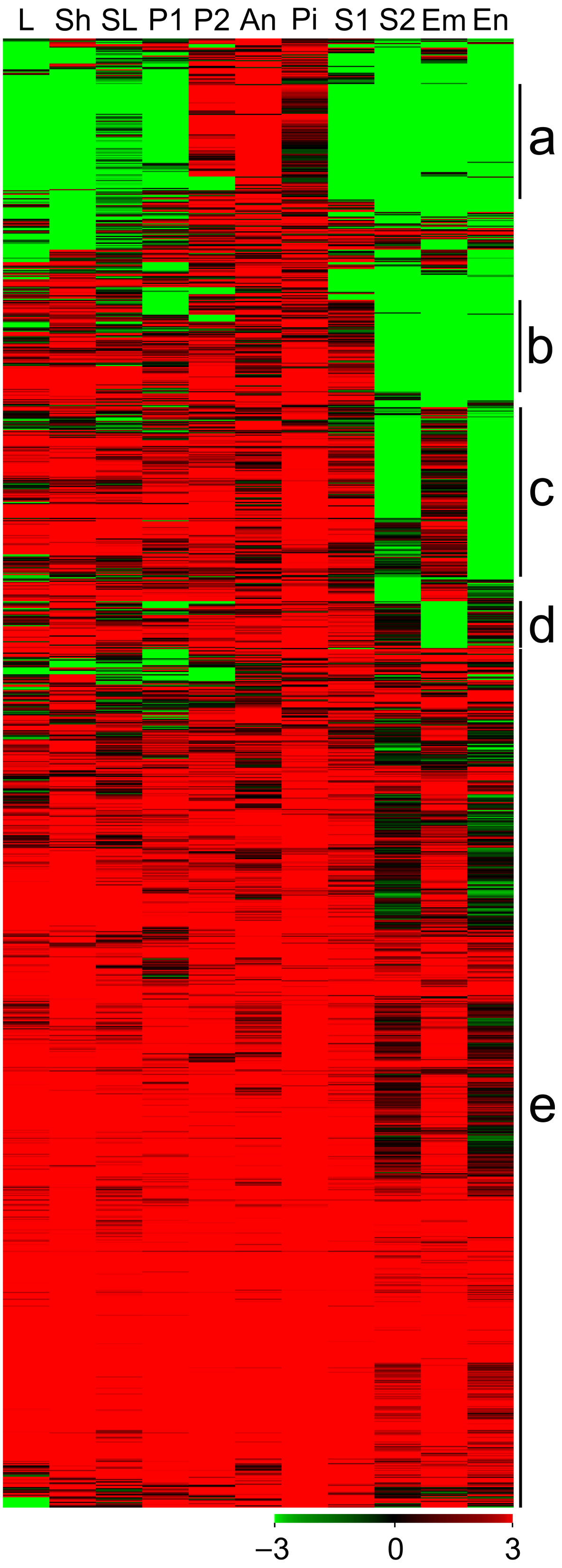

Figure S3. Heat map of the 1287 PTRGs expressed in both pistil and anther in rice. (**a**) The 100 genes were predominantly expressed in P2, the anther, and the pistil. (**b**) The 81 genes were not

expressed in seed at 10 DAP, embryo and endosperm at 25 DAP. (**c**) The 149 genes were not expressed in seed at 10 DAP and endosperm at 25 DAP. (**d**) The 41 genes were not expressed in embryo at 25 DAP. (**e**) Most of the rest genes were widely expressed in vegetative and reproductive tissues. L, 20-day-old leaf; Sh, 14-day-old seedling on the ground (shoot); SL, seedling at four-leaf stage; P1, panicle before emerging from the sheath of the flag leaf; P2, panicle after emerging from the sheath of the flag leaf; An, mature anther; Pi, mature pistil before pollination; S1, seed at 5 DAP; S2, seed at 10 DAP; Em, embryo at 25 DAP; En, endosperm at 25 DAP.

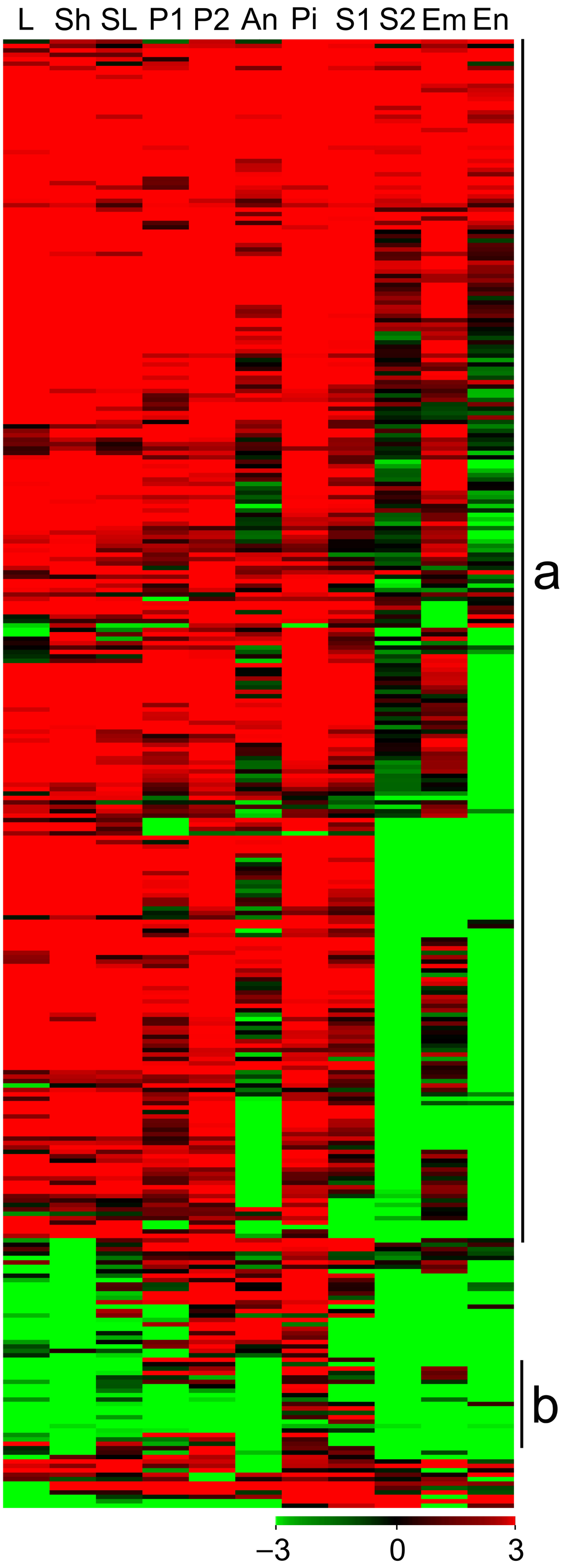

Figure S4. Heat map of the 332 genes involved in the recognition and fusion of the male-female gametes in rice. (**a**) Most genes were widely expressed in vegetative and reproductive tissues. (**b**)

The 21 genes were mainly expressed in pistil and seed at 5 DAP. L, 20-day-old leaf; Sh, 14-day-old seedling on the ground (shoot); SL, seedling at four-leaf stage; P1, panicle before emerging from the sheath of the flag leaf; P2, panicle after emerging from the sheath of the flag leaf; An, mature anther; Pi, mature pistil before pollination; S1, seed at 5 DAP; S2, seed at 10 DAP; Em, embryo at 25 DAP; En, endosperm at 25 DAP.

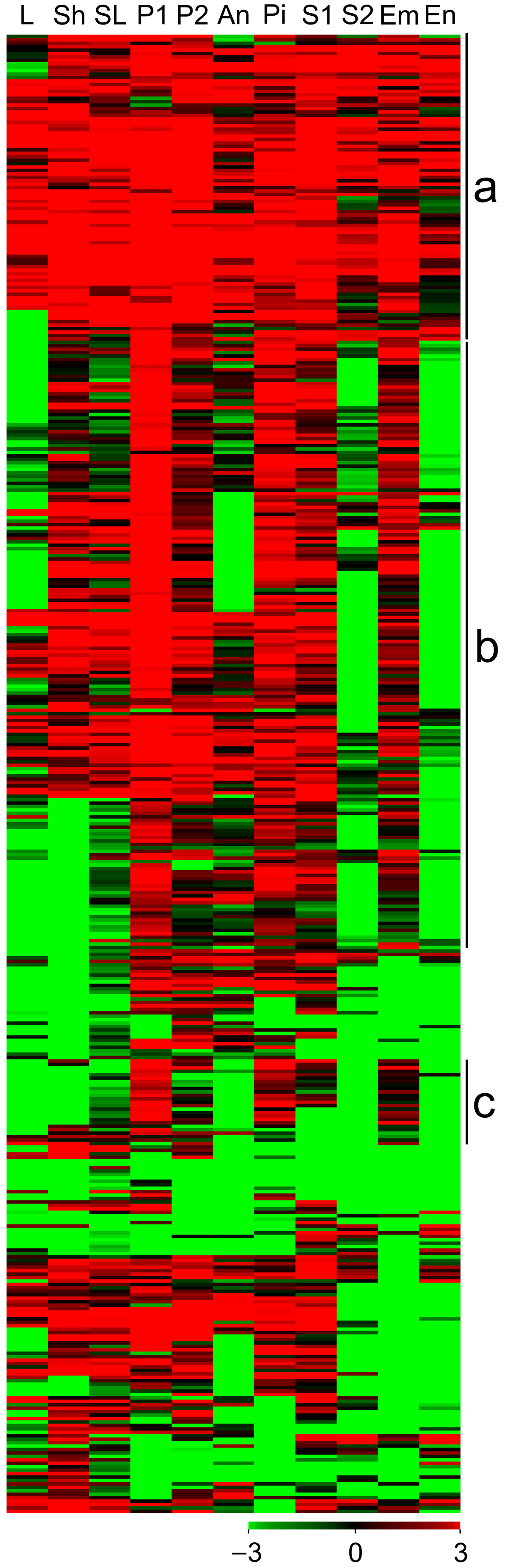
L Sh SL P1 P2 An Pi S1 S2 Em En
a
b
c
−3 0 3

Figure S5. Heat map of the 430 genes involved in zygote formation and endosperm nuclear division in rice. (**a**) About one fifth of the genes were widely expressed in vegetative and reproductive tissues. (**b**) About half of the genes were predominantly expressed in tissues other than seed at 10 DAP and endosperm at 25 DAP. (**c**) 25 genes were mainly expressed in P1, pistil and embryo at 25 DAP. L, 20-day-old leaf; Sh, 14-day-old seedling on the ground (shoot); SL, seedling at four-leaf stage; P1, panicle before emerging from the sheath of the flag leaf; P2, panicle after emerging from the sheath of the flag leaf; An, mature anther; Pi, mature pistil before pollination; S1, seed at 5 DAP; S2, seed at 10 DAP; Em, embryo at 25 DAP; En, endosperm at 25 DAP.
